# Peripheral Routes to Neurodegeneration: Passing Through the Blood–Brain Barrier

**DOI:** 10.3389/fnagi.2020.00003

**Published:** 2020-02-04

**Authors:** Patrizia Giannoni, Sylvie Claeysen, Francesco Noe, Nicola Marchi

**Affiliations:** ^1^Laboratoire CHROME (EA 7352), Université de Nîmes, Nîmes, France; ^2^CNRS, INSERM U1191, Institut de Génomique Fonctionnelle, University of Montpellier, Montpellier, France; ^3^HiLIFE – Neuroscience Center, University of Helsinki, Helsinki, Finland

**Keywords:** blood–brain barrier, neurodegeneration, peripheral immunity, traumatic brain injury, status epilepticus, autoantibodies, gut–brain axis, inflammation

## Abstract

A bidirectional crosstalk between peripheral players of immunity and the central nervous system (CNS) exists. Hence, blood–brain barrier (BBB) breakdown is emerging as a participant mechanism of dysregulated peripheral–CNS interplay, promoting diseases. Here, we examine the implication of BBB damage in neurodegeneration, linking it to peripheral brain-directed autoantibodies and gut–brain axis mechanisms. As BBB breakdown is a factor contributing to, or even anticipating, neuronal dysfunction(s), we here identify contemporary pharmacological strategies that could be exploited to repair the BBB in disease conditions. Developing neurovascular, add on, therapeutic strategies may lead to a more efficacious pre-clinical to clinical transition with the goal of curbing the progression of neurodegeneration.

## Brain Barriers’ Paths, Leaks, and Neurodegeneration

The term neurodegenerative describes a progressive deterioration of the central nervous system (CNS) that is frequently associated with abnormal accumulation of proteins. Importantly, neurofibrillary tau-protein tangles are not only a major sign of Alzheimer’s disease (AD) but are reported in temporal lobe epilepsy and post-traumatic encephalopathies (Tai et al., 2016). Among the emerging disease mechanisms, a peripheral–CNS pathological interplay is proposed to contribute to the neurodegenerative process (Marchi et al., 2014; Engelhardt et al., 2017; Fung et al., 2017; Pavlov and Tracey, 2017; Prinz and Priller, 2017; Le Page et al., 2018). Accordingly, harmful events occurring at the cerebrovascular interface are being examined as key determinants partaking to or even preceding neurodegeneration (Zlokovic, 2011; Nation et al., 2019; Sweeney et al., 2019). At the cerebrovasculature, specialized endothelial cells, mural cells, and astroglia constructs (Abbott et al., 2010; Giannoni et al., 2018; Sweeney et al., 2019) provide physical and biological properties governing the homeostatic–immune interactions between peripheral blood cells, or molecules, and brain neuroglia. The physiological parenchymal milieu composition ensures a healthy neuronal transmission, attainable because of the tightness of the blood–brain barrier (BBB; Zlokovic, 2008; Giannoni et al., 2018; Nation et al., 2019). At the pial arterial and venous level, the cerebrovasculature is permissive to blood cells or molecules, while it becomes impermeable at the arteriole–capillary level where barriers’ properties are fully established (Abbott et al., 2010). BBB vessels also contribute to cerebrospinal and interstitial fluid movements and the elimination of waste products from the interstitial and perivascular spaces (Noé and Marchi, 2019).

**FIGURE 1:**
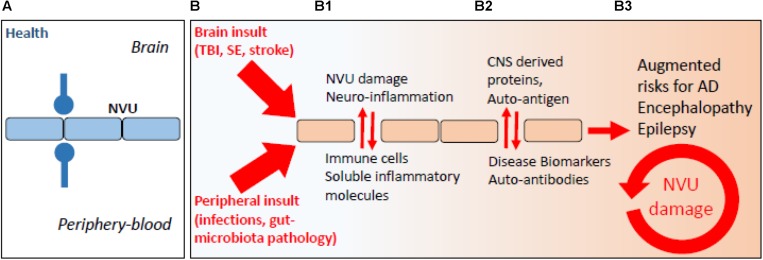
The periphery–brain interplay and CNS disease: the neurovascular pathological imprint. **(A)** Proper peripheral–brain segregation under healthy conditions (neurovascular unit, NVU; *blue lines*). **(B)** Pathological insult(s) elicited in the periphery or in the brain (traumatic brain injury, TBI; status epilepticus, SE) converge to NVU damage (e.g., BBB permeability) and neuro-inflammation, leading to temporary or prolonged loss of brain homeostatic control (**B1**, *red arrows*). **(B2)** Under conditions of BBB permeability, concentration gradients favor brain-derived proteins to extravasate into the peripheral blood. Under this condition, a peripheral auto-immune reaction may mount leading to the production of autoantibodies, possibly re-entering into the CNS if BBB damage endures **(B3)**.

It is increasingly recognized that a BBB pathological imprint can provoke a brain pro-inflammatory disequilibrium sufficient to modify neuronal activity in the long term (Marchi et al., 2007, 2014; Nation et al., 2019). Vascular-dependent mechanisms of neurodegeneration can rapidly elicit as a consequence of peripheral infections, head trauma, ischemic stroke, or status epilepticus (Figure 1; Nation et al., 2019; Sweeney et al., 2019). These are risk factors for the development of long-term neurodegenerative sequelae and encephalopathies (e.g., post-concussion or head trauma-related chronic traumatic encephalopathy, CTE), cerebral amyloid angiopathy (CAA), AD, and epilepsy. Under conditions of increased BBB permeability, an aberrant bidirectional exchange between the neurovascular unit and the peripheral blood occurs, compounding to neurodegenerative modifications (Figure 1; Marchi et al., 2014; Engelhardt et al., 2017; Fung et al., 2017; Pavlov and Tracey, 2017; Prinz and Priller, 2017; Le Page et al., 2018). Completing a vicious cycle, beta-amyloid deposition in the brain can provoke capillaries dysfunction (Thomas et al., 1996; Zhang et al., 1997; Iadecola et al., 1999; Deane et al., 2003, 2012; Nortley et al., 2019). As an example, reactive oxygen species and endothelin-1 production were proposed to elicit vasoconstriction at pericyte locations (Nortley et al., 2019). A question remains regarding whether the endothelin-1 mechanism can directly drive neurodegeneration.

## Autoantibodies and Neurodegeneration: Bad, Good, or Nil?

The communication between the peripheral blood and the brain occurs at preferential cerebrovascular sites (Zlokovic, 2011; Noé and Marchi, 2019), e.g., at post-capillary venules or pial vessels, and by a system of lymphatic vessels draining the cerebrospinal and interstitial fluids to cervical lymph nodes (Aspelund et al., 2015; Louveau et al., 2015a, b, 2018). At the intravascular level, moving leukocytes shape a peripheral–brain immune dialog where endothelium activation or permeability, perivascular immune cell homing, and brain entry of immune soluble factors prompt and sustain neuroglia inflammation [Figure 1; see Engelhardt et al. (2017) and Ransohoff (2016) for fundamental aspects of endothelial–leukocyte adhesion]. The implication of the cerebrovascular interface to innate and adaptive modalities of immunity is central (Schwartz and Shechter, 2010; Sommer et al., 2017). Adaptive immunity to the brain requires T- and B-cell stimulation at extra-CNS lymphatic organs and by professional antigen-presenting cells (Janeway et al., 2001), thus implying the existence of a peripheral–brain immune dialog, e.g., via the CNS vascular and lymphatic routes (Noé and Marchi, 2019).

A question exists on whether neurodegeneration may result from autoimmune-like processes (Table 1). Contingent to a prolonged or recurrent BBB permeability, specific antigens could exit the brain to reach the bloodstream, mounting a peripheral humoral response. Newly formed brain-directed autoantibodies could be neuropathological upon their entry into the brain across a continuously damaged BBB (Levin et al., 2010). Importantly, autoantibodies and autoreactive T cells were reported in the cerebrospinal fluid (CSF), sera, as well as in the brain of AD patients and experimental models of disease (Table 1; Kronimus et al., 2016; Wu and Li, 2016). Anti-Aβ antibodies (Ig type G) correlated with scores of dementia (Wilson et al., 2009). Intrathecal antibodies against tau filaments were reported in AD patients (Mruthinti et al., 2004) and were proposed as contributors of disease progression (Bartos et al., 2012). Anti-tau autoantibodies are not specific to AD as they are increased in patients suffering from other neurodegenerative diseases, e.g., multiple sclerosis (Fialová et al., 2011).

**TABLE 1:** Autoantibodies reported in neurodegenerative disease and post-TBI.

Autoantibodies	Neuro- pathology	Stage	Model investigated	Sample	Observed effects	Isotype	References
Anti-neuronal antibody	TBI	Moderate acute TBI	AM	Serum	–	IgG	Rudehill et al., 2006
Anti-neurofilament	AD	Moderate forms of AD	H	Serum, CSF	–	IgG. IgM	Bartos et al., 2012
Anti-Aβ	AD	Mild to severe forms of AD, early and late onset	H	Serum, CSF	Suggested to favor Aβ clearance; correlation with global scores of dementia	IgG {IgG2}, Nab	Myagkova et al., 2001; Bell et al., 2010; Daneman et al., 2010; Schwartz and Shechter, 2010; Armulik et al., 2017; Kisler et al., 2017; Rustenhoven et al., 2017; Sommer et al., 2017; Montagne et al., 2018; Nikolakopoulou et al., 2019
Anti-Tau	AD, TBI	Mid to severe forms of AD	H	Serum, CSF, tissue	Levels correlated with reduced Plaque burden	IgG, Nab	Du et al., 2001; Weksler et al., 2002; Mruthinti et al., 2004; Brettschneider et al., 2005; Rosenmann et al., 2006; Gruden et al., 2007; Gustaw et al., 2008; Britschgi et al., 2009; Wilson et al., 2009; Maftei et al., 2013; Qu et al., 2014
Anti-AMPA receptor	AD, TBI	Moderate to severe AD Mild and repetitive concussion in children	H	Serum	Levels increased in moderate and severe dementia	–	Goryunova et al., 2007
Anti-NMDA receptor	AD, TBI	Moderate to severe AD and dementia, mild and repetitive concussion	H	Serum	Relationship between autoantibody titers and aging	IgG	Goryunova et al., 2007; Busse et al., 2014
Anti-acetyl choline receptor	TBI	TBI to different severity in children	H	Serum	Levels correlate with trauma severity	–	Sorokina et al., 2011
Anti-Dopamine	AD	Mid to severe forms of AD	H	Serum	Match to moderate to severe dementia progression	IgG	Myagkova et al., 2001; Gruden et al., 2007
Anti-5-HT	AD	Mild to severe forms of AD	H	Serum	Levels increased during mild dementia	–	Myagkova et al., 2001
Anti-GFAP	AD, TBI	Pre-senile and senile forms of AD, senile vascular dementia	H	Serum	Relationship between autoantibody titers and aging suggested as a maker of BBB damage	IgG	Tanaka et al., 1989; Gruden et al., 2007
Anti-S100β	AD, TBI	Mild to severe AD, senile vascular dementia, repealed acute sub-concussion	H	Serum	Match to moderate–severe dementia progression; relationship between autoantibody titers and aging	IgG	Mecocci et al., 1995; Gruden et al., 2007; Marchi et al., 2013
Anti-microglia	AD	Mid to severe forms of AD	H	CSF	–	IgG	McRae et al., 2007
Anti-phospholipid	AD, TBI	Mid cognitive impairment to advanced AD, severe TBI	H	Serum, CSF	Levels correlate with erythrocytes and proteins in CSF	IgG	McIntyre et al., 2007; McIntyre et al., 2015
Anti-ceramide	AD	Chronic pathology in TG mice	AM	Serum	Levels correlate with plaque formation	IgG	Dinkins et al., 2015
Anti-RAGE	AD	Mild cognitive impairment to severe AD	H	Serum	Relationship with anti-Aβ levels; correlation with global scores of dementia	IgG	Mruthinti et al., 2004; Wilson et al., 2009
Anti-ATP synthase	AD	Mild to severe AD	H	Serum	Induced the inhibition of ATP synthesis	IgG	Vacirca et al., 2012
Anti-pituitary	TBI	Mild to severe TBI, acute and long-term	H	Serum	Association between antibody positivity and hypopituitarism due to head trauma	IgG	Tanriverdi et al., 2008; Tanriverdi et al., 2010; Pani et al., 2019
Anti-adrenergic receptors	AD	Mild to moderate dementia	H	Serum	Suggested contribution to vascular lesions and increased plaque formation	IgG	Karczewski et al., 2012

*AM, data derives from animal models only.*

The significance of peripheral autoantibodies as biomarkers of neurodegenerative conditions also remains to be established. Autoantibodies against the glutamate receptor *N*-methyl-D-aspartate receptor (NMDAR) were detected in plasma of AD patients (Davydova et al., 2007). Levels of antibodies were shown to correlate with clinical severity, as patients affected by moderate and severe dementia presented a twofold autoantibody increase compared with patients suffering from mild dementia (Davydova et al., 2007). The presence of autoantibodies against 5-HT was also reported (Myagkova et al., 2001), with levels increasing during the mild phase of the disease, subsequently reaching a plateau (Myagkova et al., 2001). Similar findings were reported for autoantibodies directed against the receptor for advanced glycation end products (Wilson et al., 2009). In a transgenic model of AD, autoantibodies against the sphingolipid ceramide correlated with amyloid plaque increase (Posse de Chaves and Sipione, 2010; Dinkins et al., 2015). Autoantibodies against ATP synthase (Vacirca et al., 2012), α(1)-adrenergic, and the β(2)-adrenergic receptors were also reported (Karczewski et al., 2012). Autoantibodies against the α(1)-adrenergic and the β(2)-adrenergic receptors may contribute to vascular lesions and increased plaque formation in AD patients (Karczewski et al., 2012).

Importantly, not all autoantibodies are harmful. Brain-reactive natural autoantibodies (NAbs) are protective (Britschgi et al., 2009; Kellner et al., 2009; Dodel et al., 2011; Bach and Dodel, 2012). NAbs are mostly IgM and are spontaneously produced. NAbs are polyreactive with low affinity for self-antigens (Casali and Schettino, 1996). Physiologically, NAbs facilitate phagocytosis of apoptotic cells, inhibit inflammatory pathways, and have a role in maintaining immune tolerance (Elkon and Silverman, 2012). NAbs to Aβ can inhibit plaque aggregation, block Aβ toxicity, and catalyze Aβ clearance (Lindhagen-Persson et al., 2010). Immunotherapies using specific, or aspecific, autoantibodies were tested. Bapineuzumab is the humanized form of a monoclonal anti-Aβ antibody targeting the N-terminus of Aβ. In phase II trials, Bapineuzumab administration reduced Aβ plaques in AD brains (Salloway et al., 2009; Rinne et al., 2010) and was associated with decreased total and phospho-tau levels in the CSF (Asuni et al., 2007). Bapineuzumab was, however, discontinued after a phase III trial and showed no beneficial effects on cognitive or functional outcomes (U.S. National Library of Medicine, 2019a, b). Aducanumab (BIIB037) is a human monoclonal antibody selectively targeting aggregated Aβ (oligomers and fibrils) (Sevigny et al., 2016). An Aducanumab phase III trial was terminated as endpoints were not meet. The analysis of a larger data set is ongoing. Tau immunotherapies are also being developed, attenuating or preventing functional impairment in experimental models, as reviewed in Sigurdsson (2018).

## Autoantibodies and Post-Traumatic Encephalopathy

Resulting from repeated head trauma and BBB damage, chronic traumatic encephalopathy (CTE) presents with accumulation of neurofibrillary tau-protein tangles. In TBI subjects, blood and CSF autoantibodies were suggested as etiological components or as possible biomarkers of neurodegeneration (Raad et al., 2014; Kobeissy, 2015; Table 1). Anti-glial fibrillary acidic protein (GFAP) fragments were found in the sera of TBI patients (Zhang et al., 2014). Serum autoantibodies against S100B were reported in American football players when repeated sub-concussive events were associated with BBB damage (Marchi et al., 2013). Autoantibodies against the neuronal α7-subunit of the acetylcholine receptor (Sorokina et al., 2011) as well as AMPA and NMDA receptors (Goryunova et al., 2007) were detected in TBI subjects, while IgG autoantibodies to neurons and basal lamina were reported in rat serum following experimental head trauma (Rudehill et al., 2006). Autoantibodies to the pituitary gland were reported in TBI subjects 3 years after the trauma (Tanriverdi et al., 2008, 2010). Damage to the pituitary gland is distinctive of the TBI pathology with 20–50% of patients showing some degrees of pituitary dysfunction, which affects growth hormone production (Aimaretti et al., 2005; Tanriverdi et al., 2006). An association between anti-pituitary autoantibodies and pituitary dysfunction was reported in patients suffering from mild TBI, including repetitive concussions (Tanriverdi et al., 2010).

Autoreactive antibodies have been proposed for the treatment of TBI sequelae. The presence of hyper-phosphorylated tau accumulating in neurofibrillary tangles is a characteristic of CTE (Omalu et al., 2010). Even if phospho-tau is detectable only at low levels acutely after TBI (Smith et al., 2003; Blennow et al., 2012; Goldstein et al., 2012; Mannix et al., 2013), a specific form of phospho-tau can be produced in response to TBI (*cis* P-tau) (Kondo et al., 2015). This protein spreads throughout the brain, harming cells and leading to post-traumatic neurodegeneration and dementia. In two animal models of TBI, administration of a monoclonal antibody discriminating between the *cis* and the *trans* forms of the protein and blocking *cis* P-tau prevented the onset of tauopathy and cortical atrophy. These accumulating evidence supports the possible involvement of autoantibodies in post-TBI neurodegenerative conditions, perhaps providing new disease biomarkers and therapeutic entry points.

## The Gut–Brain Axis and Neurodegeneration: Is There a Barrier Implication?

**FIGURE 2:**
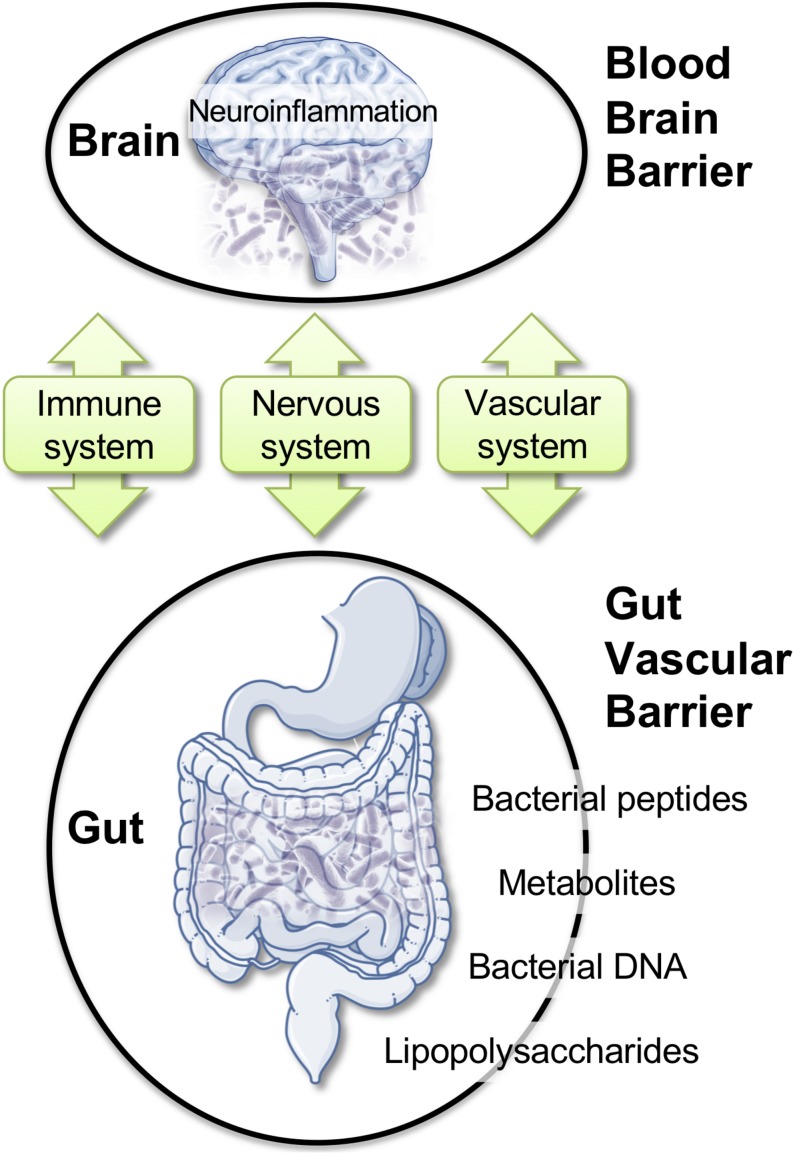
Gut–brain axis: communication routes and physiological barriers. A double, peripheral, and brain homeostatic control is performed by the intestinal–epithelial and blood–brain barriers under healthy conditions. Rupture of one barrier (e.g., gut) may impact the other (e.g., brain), with the blood stream and the immune system being the facilitators or the arbitrators of the pathological spread and neuro-inflammation.

Here, we discuss a specific framework where alterations of the gut microbiota (GM) could impact BBB permeability, promote neuro-inflammation, and favor neurodegenerative modifications (Figure 2; Braniste et al., 2014; Cerovic et al., 2019; Parker et al., 2019; Wang et al., 2019). Bacteria, viruses, parasites, and non-pathogenic fungi constitute the intestinal microbiota. These complex communities of microbes colonizing the gastrointestinal tract are major players in health. Modern life and diets have progressively induced changes in the composition of the GM, perhaps for the worse, as this can contribute to chronic illnesses (Lozupone et al., 2012; Myles, 2014; Kumar and Forster, 2017; Shanahan et al., 2017; Cryan et al., 2019; Pagliai et al., 2019; Reza et al., 2019). Intestinal microbes can influence brain function through a continuous dialog involving the immune, the vascular, and the nervous systems (Figure 2; Schroeder and Bäckhed, 2016; Cox and Weiner, 2018; Butler et al., 2019; Cryan et al., 2019). Modifications in the composition of the GM was reported in brain disorders, such as autism (Adams et al., 2011; Kang et al., 2019), depression (Kelly et al., 2016; Zheng et al., 2016), Parkinson’s disease (Scheperjans et al., 2015; Sampson et al., 2016), and AD (Cattaneo et al., 2017; Vogt et al., 2017; Zhuang et al., 2018). Intriguingly, the extent of the amyloid pathology in AD mice appears to be dependent of the microbial status, which is specific to the animal housing facility. APP/PS1 mice bred in a germ-free facility displays decreased amyloid plaque number compared to mice housed in non-germ-free conditions (Harach et al., 2017). Moreover, the administration of broad-spectrum, combinatorial antibiotics to APP/PS1 mice, either during the peri-natal or the adult stage, reduced brain Aβ deposition (Minter et al., 2016, 2017).

Existing reports support the hypothesis of a possible infectious origin of AD. Aβ was proposed as an antimicrobial peptide (Soscia et al., 2010; Moir et al., 2018) responding to pathogens (Kumar et al., 2016; Eimer et al., 2018). Infectious agents, such as *Chlamydia pneumonia*, *Proprionibacterium acne*, *Helicobacter pylori*, *Porphyromonas gingivalis*, or *spirochetes*, are associated with AD (Kornhuber, 1996; Balin et al., 1998; Kountouras et al., 2006; Miklossy, 2011; Poole et al., 2015). A microbial hypothesis is supported by evidence describing the presence of viruses, such as Herpes simplex virus type I, in the brains of AD patients (Lin et al., 2002; Alonso et al., 2014; Itzhaki et al., 2016).

Within the complex interplay between the gut microbiome and the CNS, a role for brain barriers and neuroinflammation is becoming important (Braniste et al., 2014; Cerovic et al., 2019; Parker et al., 2019; Wang et al., 2019). The impact of the gut microbiome composition on CNS health was reported (Amedei and Boem, 2018; Chu et al., 2019; Sherwin et al., 2019; Virtue et al., 2019). Recent work demonstrated that GM composition controls BBB development and permeability in mice (Braniste et al., 2014). In AD, increased gut permeability due to GM dysbiosis was reported during prolonged stress. In this condition, molecules that are normally secluded in the intestine, e.g., inflammatory mediators, bacteria, or bacterial-derived agents, could leak out and reach the peripheral blood. Bacterial DNA, metabolites, or proteins circulating in the blood stream could, in turn, modify BBB permeability (Braniste et al., 2014; Myles, 2014; Kumar and Forster, 2017; Cerovic et al., 2019; Parker et al., 2019; Wang et al., 2019). Existing reports indicated bacterial DNA in human blood with a possibility for brain access (Lelouvier et al., 2016; Païssé et al., 2016; Schierwagen et al., 2018). Brain entry of *P. gingivalis*, a bacterium associated with periodontal disease, has been described (Dominy et al., 2019). Gingipain inhibitors reduced the bacterial load and the bacteria-induced neuro-inflammation in a mouse model (Dominy et al., 2019). Among Spirochetes, *Borrelia burgdorferi* is a strain associated with Lyme dementia that could enter the brain. In humans, this specific strain can form biofilms similar to senile plaques. Aβ and bacterial DNA appear as important constituents of these biofilms, suggesting that amyloid plaques may originate in association with or from the spirochetal colonies (Allen, 2016; Miklossy, 2016).

These examples highlight the need of tightly regulated intestinal and brain barriers (Rahman et al., 2018). In AD, a dysbiotic GM may enhance gut permeability and alter BBB integrity, allowing the access of infectious agents or associated molecules into the brain (Martin et al., 2018). Significantly, intestinal and brain barriers are reactive to analogous pro-inflammatory triggers. Circulating inflammatory cytokines IL-17, interferon-gamma (IFN-γ), and the small intestine epithelium protein zonulin can damage the intestinal–epithelia and BBBs (Rahman et al., 2018).

## Gut Microbiota and Autoantibodies: Initial Clues

Hypotheses linking modifications of the GM and production of autoantibodies are emerging (Petta et al., 2018). Some evidence supports the concept that specific dietary components may affect B-cell maturation and activity, ultimately leading to the formation of autoantibodies (Petta et al., 2018). Obesity was associated with a systemic pro-inflammatory state, characterized by changes in the frequency of B-cell subpopulation [e.g., reduction of the anti-inflammatory IL-10^+^ regulatory B cell (Nishimura et al., 2013)] and by an increase in autoantibody levels (Kosaraju et al., 2017). Diets rich in polyunsaturated fatty acid are associated with the suppression of pro-inflammatory responses and a reduction of circulating autoantibodies (Pestka et al., 2014; Tomasdottir et al., 2014). Dietary components impact the composition of the gastrointestinal bacterial populations: consumption of prebiotics increases the intestinal levels of *Bifidobacterium* and *Lactobacillus* (Singh et al., 2017), with a possible link to B-cell differentiation, maturation, and activation (Ouwehand et al., 2002). Diet can impact autoantibody production, directly by promoting pro-inflammatory conditions and indirectly by altering the GM. In experimental autoimmune encephalomyelitis (EAE) it was demonstrated that the commensal microbiota composition is a pivotal factor for disease development (Lee et al., 2011) and that modifying the GM impacts the levels of T and B cells or the levels of circulating autoantibodies (Ochoa-Repáraz et al., 2009, 2010).

## BBB Repairing Pharmacology: Available Options

The multi-level implication of BBB damage in neurodegenerative disorders has prompted the quest for pharmacological repairing strategies, either directed at the endothelium or by indirect targeting of the cellular players of peripheral and neuro-inflammation. Currently tested drugs are either repurposed or new (Table 2). Examples include losartan, an anti-hypertensive molecule acting as an angiotensin II antagonist. Losartan was shown to reduce BBB permeability in a rat model of hypertension (Kucuk et al., 2002; Kaya et al., 2003) and following pilocarpine-induced status epilepticus (Hong et al., 2019). BBB protection by losartan depends on angiotensin receptor type 1 (AT1) blockade. Another drug is rapamycin, a specific inhibitor of the mammalian target of rapamycin (mTOR) pathway. Rapamycin improved cerebrovascular and cognitive function in a mouse model of AD (Van Skike et al., 2018). Inhibition of mTOR preserved BBB integrity through the upregulation of tight junction proteins and downregulation of matrix metalloproteinase-9. A third option is anakinra, which is the recombinant form of the human IL-1 receptor antagonist (IL1-Ra) that inhibits IL-1α and IL-1β binding to the IL-1 receptor type 1. As inflammation comprises BBB dysfunction, the inhibition of IL-1 as proposed is a strategy enabling cerebrovascular protection (Marchi et al., 2009, 2011; Vezzani et al., 2011; Kenney-Jung et al., 2016). Recent strategies include the development of IL-1Ra molecules fused with a cell-penetrating peptide to enhance brain access (Zhang et al., 2017). After transient middle cerebral artery occlusion in rats, IL-1Ra-PEP reduced neuro-inflammation and ischemia (Zhang et al., 2017). The fourth option is IPW-5371, a small molecule blocking the transforming growth factor β receptor (TGFβR) signaling. In a recent study (Senatorov et al., 2019), IPW reduced hyperexcitability in a mouse model, protecting BBB functions. The activated protein C (APC) therapeutic analog 3K3A-APC is a fifth option. This compound has BBB and neuro-protective properties (Thiyagarajan et al., 2008; Zhong et al., 2009; Wang et al., 2016; Sinha et al., 2018; Lazic et al., 2019; Lyden et al., 2019) and it is in clinical trial for stroke treatment (Lyden et al., 2019). Next is platelet-derived growth factor subunits BB (PDGF-BB). Following an acute vascular insult, activation of the PDGF receptor beta (PDGFRβ) by PDGF-BB is beneficial, protecting the endothelium–pericyte structures. The latter was reported in mouse models of status epilepticus (Arango-Lievano et al., 2018) and cerebral ischemia (Marushima et al., 2019). Conversely, in chronic disease settings (e.g., AD, epilepsy, etc.), activation of PDGFRβ may participate to inflammation (Rustenhoven et al., 2017; Klement et al., 2019). Under this circumstance, blocking PDGFRβ signaling by using the tyrosine kinase inhibitor Imatinib could represent an anti-inflammatory strategy (Rustenhoven et al., 2017; Klement et al., 2019). In general, reducing PDGFRβ signaling could lead to contrasting effects, e.g., pericyte deficiency and BBB breakdown (Bell et al., 2010; Daneman et al., 2010; Armulik et al., 2017; Kisler et al., 2017; Montagne et al., 2018; Nikolakopoulou et al., 2019) or anti-inflammatory (Rustenhoven et al., 2017; Klement et al., 2019), depending on disease stage (acute vs. chronic). Another option, Dexamethasone, is a glucocorticoid effective in the formation and maintenance of endothelial tight junctions (Hue et al., 2015; Na et al., 2017). Dexamethasone was proposed to decrease the expression of the Jumonji Domain Containing 3 gene (JMJD3) and metallo-proteinases (MMP-2, MMP-3, and MMP-9). Finally, there is the vascular endothelial growth factor (VEGF). Amyloid accumulation is associated with endothelial apoptosis (Religa et al., 2013) in Alzheimer’s patients as well as in mouse models. In AD mice, VEGF administration rescued memory deficits by preventing Aβ-induced vascular apoptosis (Religa et al., 2013). See Table 2 for complete drug listing, mechanisms and bibliography.

**TABLE 2:** Available molecules exerting BBB repairing and anti-inflammatory effects.

	Category	Mechanism(s) of action	Reported effects	*In vivo* /*in vitro* models	Clinical trials	References
Losartan	Antihypertensive	Angiotensin II antagonist	Improves barrier function	Rats	Antihypertensive drug	Kucuk et al., 2002; Kaya et al., 2003; Hong et al., 2019
Ripamycin	Immunosuppressant	mTOR antagonist	Improves barrier function, promotes claudin-5	Mice	Prevention of transplant rejection	Van Skike et al., 2018
Anakinra	lnterleukin-1 receptor antagonist	Human interleukin-1 receptor antagonist (IL-IRa)	Decreases inflammation	Rats	Anti-inflammatory drug currently used against rheumatoid arthritis cryopyrin-associated periodic syndromes (CAPS) and Still’s disease.	Kenney-Jung et al., 2016; Van Skike et al., 2018
IPW	TGFβR1 kinase inhibitor	Inhibition of TGFβR signaling	Reduces brain hyperexcitability, indirect BBB repair	Mice	NA	Senatorov et al., 2019
3K3A-APC	Activated protein C (APC)	BACE-1 inhibition, activation of protease-activated receptor 1	Cytoprotective properties, neovascularization, neurogenesis	Mice	In clinical trial for ischemic stroke (RHAPSODY)	Thiyagarajan et al., 2008; Zhong et al., 2009; Wang et al., 2016; Sinha et al., 2018; Lazic et al., 2019; Lyden et al., 2019
PDGF-BB	PDGFRb agonist	Increased expression of p-Smad2/3	Ameliorates BBB function	*In vivo*	NA	Arango-Lievano et al., 2018
Imatinib	Kinase inhibitor	Inhibition of PDGFR signaling	anti-inflammatory?	Mice	Precursor cell lymphoblastic leukemia–lymphoma, dermatofibrosarcoma	Su et al., 2015; Klement et al., 2019
Dexamethasone	Glucocorticoid	Decreased JMJD3 gene expression, suppression of MMP-2, MMP-3, and MMP-9 gene activation	Preserves tight junctions integrity	*In vitro* BBB model	Inflammatory conditions	Hue et al., 2015; Na et al., 2017
Annexin-A1 (ANXA1)	Glucocorticoid anti-inflammatory effector	Binding to FRP2 receptor, inhibition of phospholipase-2	Restores cell polarity, cytoskeleton integrity, and paracellular permeability	*In vitro* BBB model, Anxa−/− mice	NA	Cristante et al., 2013; Purvis et al., 2019; Zub et al., 2019
VEGF	Vascular endothelial growth factor	Prevention of Aβ-induced apoptosis	Improves vascular functions	Mice	NA	Religa et al., 2013
Tetramethylpyrazine	Cardiovascular	Blocking JAK/STAT signaling	Reduces BBB damage	Rats	NA	Gong et al., 2019
*S*-nitrosoglutathione	Nitric oxide donor	Suppression of MMP-9 activity	Prevents BBB damage	Mice	NA	Aggarwal et al., 2015
Cannabidiol	Analgesic, anti-inflammatory, antineoplastic	Activation of PPARy and 5-HT_1A_ receptors	Prevents BBB damage	*In vitro* BBB model	NA	Hind et al., 2016

## Perspectives and Challenges

The importance of cerebrovascular dysfunction in neurodegenerative disorders is twofold: BBB damage is pathophysiological and it allows a diagnostic window, the latter by exploiting specific proteins that shed from the damaged or vascular wall cells to appear into accessible fluids, e.g., blood or CSF. For instance, by dosing soluble PDGFRβ in CSF and by using dynamic contrast-enhanced magnetic resonance imaging, a recent study demonstrated BBB breakdown as an early biomarker of human cognitive dysfunction (Montagne et al., 2015; Nation et al., 2019).

Tackling the complex neurodegenerative puzzle requires a continuous sharpening of pharmacological tools. This is important because no efficacious disease-modifying strategy is available to meaningfully delay or prevent disease progression. The problematics here presented may stem from semantic habits as the term *neuro-* indicates, for most, neurons only. Revisiting nomenclature(s) may benefit, if not legitimize, holistic, and neurovascular approaches to CNS disorders since it is evident that considering neuronal circuits insulated from the influence of glio-vascular cells is excessively reductionist.

## Author Contributions

NM planned, drafted, and corrected most of the manuscript, including figures and tables. FN wrote the parts on auto-immunity and created the table. SC was responsible for the section “The Gut-Brain Axis and Neurodegeneration: Is There a Barriers’ Implication?”. PG contributed to the section on BBB drugs and to the table, and also contributed to the sections “Gut Microbiota and Autoantibodies Production: Initial Clues” and “References.”

## Conflict of Interest

The authors declare that the research was conducted in the absence of any commercial or financial relationships that could be construed as a potential conflict of interest.
